# Prospective validation of an 11-gene mRNA host response score for mortality risk stratification in the intensive care unit

**DOI:** 10.1038/s41598-021-91201-7

**Published:** 2021-06-22

**Authors:** Andrew R. Moore, Jonasel Roque, Brian T. Shaller, Tola Asuni, Melissa Remmel, David Rawling, Oliver Liesenfeld, Purvesh Khatri, Jennifer G. Wilson, Joseph E. Levitt, Timothy E. Sweeney, Angela J. Rogers

**Affiliations:** 1 Stanford Department of Medicine, Stanford, CA USA; 2 Division of Pulmonary and Critical Care Medicine, Stanford University School of Medicine, Stanford, CA USA; 3 Inflammatix, Inc., Burlingame, CA USA; 4 Institute for Immunity, Transplantation and Infections, Stanford University, Stanford, CA USA; 5 Department of Emergency Medicine, Stanford University School of Medicine, Stanford, CA USA

**Keywords:** Transcriptomics, Predictive medicine, Prognosis, Biomarkers

## Abstract

Several clinical calculators predict intensive care unit (ICU) mortality, however these are cumbersome and often require 24 h of data to calculate. Retrospective studies have demonstrated the utility of whole blood transcriptomic analysis in predicting mortality. In this study, we tested prospective validation of an 11-gene messenger RNA (mRNA) score in an ICU population. Whole blood mRNA from 70 subjects in the Stanford ICU Biobank with samples collected within 24 h of Emergency Department presentation were used to calculate an 11-gene mRNA score. We found that the 11-gene score was highly associated with 60-day mortality, with an area under the receiver operating characteristic curve of 0.68 in all patients, 0.77 in shock patients, and 0.98 in patients whose primary determinant of prognosis was acute illness. Subjects with the highest quartile of mRNA scores were more likely to die in hospital (40% vs 7%, p < 0.01) and within 60 days (40% vs 15%, p = 0.06). The 11-gene score improved prognostication with a categorical Net Reclassification Improvement index of 0.37 (p = 0.03) and an Integrated Discrimination Improvement index of 0.07 (p = 0.02) when combined with Simplified Acute Physiology Score 3 or Acute Physiology and Chronic Health Evaluation II score. The test performed poorly in the 95 independent samples collected > 24 h after emergency department presentation. Tests will target a 30-min turnaround time, allowing for rapid results early in admission. Moving forward, this test may provide valuable real-time prognostic information to improve triage decisions and allow for enrichment of clinical trials.

## Introduction

In the last two decades, utilization of the intensive care unit (ICU) has increased significantly. In one study of hospitalizations in 29 states in 2011, over one quarter of patients required ICU care during their admissions^1^. In 2010, ICU care alone in the United States cost $108 billion^2^. Accurate risk stratification tools for critically ill patients would not only enable optimal allocation of scarce and expensive ICU resources, but could also enhance prognostic and potentially predictive enrichment of clinical trials^3–5^. Several clinical scoring systems help predict ICU mortality on a population-level, but they are poorly predictive for any given individual^6,7^. The most widely known of these scoring systems are the Simplified Acute Physiology Score (SAPS) and the Acute Physiology and Chronic Health Evaluation (APACHE) score^8–10^. In addition to their limited predictive capacity, these tools are designed around ICU admission data, and therefore cannot be used prospectively to risk-stratify patients at emergency department admission or to select patients for enrollment into clinical trials^3^.

To address these shortcomings, there has been significant interest in developing molecular diagnostic assays to better risk stratify patients with critical illness^3,11–13^. For example, latent class modeling of multiple populations of acute respiratory distress syndrome (ARDS) patients has identified a high-mortality subset, defined by clinical markers of shock and proteomic plasma biomarkers of inflammation^14,15^. Several studies in septic patients have shown significant immune dysregulation at the genomic and transcriptomic level^16–19^, and a recent study led by collaborators developed a prognostic model for sepsis mortality based on whole blood messenger RNA (mRNA) expression^20^. The model was developed in 12 publicly available cohorts of community-acquired and hospital-acquired sepsis and validated in 9 additional cohorts, together encompassing more than 900 sepsis survivors and 200 non-survivors. The best-performing model was a so-called “Stanford” multi-mRNA mortality score, which was associated with an area under the receiver operating characteristic curve (AUROC) for mortality of 0.89 in validation testing.

In this work, we test the previously identified mRNA mortality score both as a standalone test and in combination with SAPS 3 and APACHE II for association with mortality in a heterogeneous, prospectively collected medical ICU population. We further examine the impact of timing of blood draw, patient comorbidities, and acuity of illness as a driver of prognosis on test performance.

## Methods

### Subjects

We collected blood into PAXgene RNA tubes from 165 patients enrolled in the Stanford University Medical ICU Biobank from 2015 to 2018. Adult subjects enriched for acute respiratory distress syndrome risk factors (e.g. sepsis, aspiration, trauma) were recruited at admission to the Stanford ICU from either the hospital wards or the emergency department as part of an existing biobanking study. Patients eligible for inclusion were consecutive adults (≥ 18 years) admitted to Stanford ICU with at least one ARDS risk factor (e.g. sepsis, pneumonia, trauma, aspiration). We excluded routine post-op patients, those admitted for a primary neurologic indication, and those with anemia (hemoglobin < 8). Screening of consecutive new admissions via electronic medical records review of all ICU subjects was performed by a study coordinator and the study principal investigator (AJR). Screening occurred on weekdays with a goal enrollment of < 24 h of ICU admission, and included patients admitted to the ICU from the wards or the emergency room. Patients or their surrogates were approached for consent to participate in the Stanford ICU biobank.

Clinical data was abstracted from the electronic medical record by study staff blinded to the 11-gene score. Data collected included patient demographics, past medical history, and all physiologic and laboratory data required to calculate SAPS 3 and APACHE II scores. All data was compiled in REDCap. This study was approved by, and all patient samples and data collected were in compliance with, the Stanford Institutional Review Board (Stanford IRB #28205). Written informed consent was obtained from all participants or their surrogates or was waived in select circumstances in accordance with IRB protocol. All methods were carried out in accordance with relevant guidelines and regulations.

### 11-gene mortality score

A previous study showed the prognostic validity of the “Stanford” multi-mRNA score in predicting the risk of mortality from sepsis at disease onset^20^. The gene score is calculated as the difference in geometric mean of the expression value of two gene ‘modules’ composed of over-expressed and under-expressed genes. The upregulated genes include *DEFA4, CD163, RGS1, PER1, HIF1A, SEPP1, C11orf74,* and *CIT*, while the down-regulated genes include *LY86, TST,* and *KCNJ2*. One previously identified gene, *OR52R1,* was removed from the 12-gene mRNA panel because a lack of introns limited later assay development and test performance was found to be similar when *OR52R1* was excluded. The resulting 11-gene mRNA score is used in this work.

De-identified clinical samples were shipped frozen to Inflammatix and run by technicians blinded to clinical outcomes. RNA was isolated from PAXgene RNA tubes with the RNeasy Plus Micro Kit (QIAGEN, Germantown, MD, part #74034) on a QIAcube (QIAGEN), using a custom protocol. Expression levels were quantitated on the NanoString nCounter (NanoString, Seattle, WA) using 150 ng of total RNA per sample hybridized for 16 h at 65 °C per manufacturer’s instructions. The nCounter SPRINT standard protocol was followed to generate mRNA counts. The raw mRNA counts were normalized across samples using the geometric mean of counts for 4 housekeeping genes (*CDIPT, KPNA6, RREB1, YWHAB),* per manufacturer instructions. The 11-gene score was calculated using the geometric mean of the genes that are up-regulated mRNAs minus 3/8 times the geometric mean of the down-regulated mRNAs.

### Statistical analysis

Demographic data were compared using the Wilcoxon rank-sum test for continuous data and Fisher’s exact test for categorical data. The primary outcome was improvement in area under the receiver operating characteristic curve (AUROC) for 60-day mortality of the 11-gene score compared to SAPS 3 and APACHE II scores in patients whose sample was obtained within 24 h of arrival to emergency department. This time cutoff was pre-selected because of a previously identified time-sensitivity of gene expression changes in early sepsis^20–22^; thus, we excluded outside hospital and floor transfers that were > 24 h from emergency department presentation. AUROC values were compared using DeLong’s test for two ROC curves^23^. We evaluated test performance using a pre-defined score cutoff at the top quartile of 11-gene score for the entire patient population.

To evaluate the relationship between the 11-gene score and survival times, Kaplan–Meier survival curves were generated and compared using the log-rank test to assess survival in patients in the top quartile vs patients in all other quartiles. We further evaluated test performance in predicting 30-day mortality as well as in-hospital mortality. Odds ratios were calculated and compared using Fisher’s exact test.

To evaluate whether the addition of the 11-gene score improved prognostication, categorical Net Reclassification Improvement (NRI) Index and Integrated Discrimination Improvement (IDI) Index were calculated using pre-defined 60-day mortality risk cutoffs of < 10%, 10–30%, 30–50%, and > 50% comparing the performance of APACHE II and SAPS 3 alone vs in combination with the 11-gene score using logistic regression modeling.

We further looked at performance in predicting both 60-day mortality as well as in-hospital mortality in two pre-specified subgroups: (1) patients in shock, which was defined as requiring at least one vasopressor, and (2) patients whose primary driver of prognosis was multi-system organ dysfunction syndrome (MODS) or ARDS. Primary driver of prognosis was determined by clinicians (A.J.R, J.E.L., or A.R.M.), blinded to 11-gene, APACHE II, or SAPS 3 scores. The roles of each of the following categories in the patient’s prognosis was scored from 1 to 5 (1 being non-contributory and 5 being highly contributory): age, goals of care, comorbidities, baseline functional status, acute multi-organ failure, ARDS, terminal illness, acute neurologic injury, and other. Each patient’s primary determinant of mortality was then grouped into one of three categories based on these result: (1) MODS or ARDS (> = 4 for ARDS or multi-organ failure), (2) Comorbidities (> = 4 for age, goals of care, comorbidities, or baseline functional status), or (3) Mixed (> = 4 for both of the above).

All statistical analysis was performed on R v3.5.1. ROC curves and NRI/IDI were generated using the PredictABEL package. Kaplan–Meier curves were generated using the survminer package.

### Ethics declarations

This study was approved by, and all patient samples and data collected were in compliance with, the Stanford Institutional Review Board (Stanford IRB #28205). Written informed consent was obtained from all participants or their surrogates or was waived in select circumstances in accordance with IRB protocol. All methods were carried out in accordance with relevant guidelines and regulations.

## Results

### Subject characteristics

Of the 165 patients with PAXgene mRNA samples, 70 (42%) samples were collected within the pre-defined 24 h emergency department cutoff time and were included in our primary analyses. Characteristics of these patients are included in Table 1. Of the patients who did not meet the cutoff time, 49 (52%) were transferred from a non-ICU ward, 30 (32%) were ED admissions with late collections, and 16 (17%) were transfers from other ICUs. Patients who met the 24 h emergency department cutoff were significantly older, had a lower incidence of shock (defined as requiring a vasopressor), and lower SAPS 3 and APACHE II scores (all p < 0.05). Despite these differences, there was no significant difference in 60-day mortality between groups with a 20% mortality in patients included in the primary analysis compared to 26% in those patients who did not meet the emergency department cutoff or were transferred to the ICU later in their disease course(p = 0.36).

**Table 1:** Patient characteristics.

	Samples collected within 24 h (n = 70)	Samples that did not meet cutoff (n = 95)	P-value
**Median age (IQR)**	71 (60–81)	64 (52–72)	0.01
**% Female**	40%	46%	0.43
**Race**			
White	52 (74%)	60 (63%)	0.18
Black/African American	4 (6%)	4 (4%)	0.72
Asian/Pacific Islander	7 (10%)	7 (7%)	0.58
Other/unknown	7 (10%)	24 (25%)	0.02
**Infection**	47 (67%)	62 (65%)	0.73
**Median SAPS 3 (IQR)**	62 (54–73)	71 (57–83)	0.01
**Median APACHE II (IQR)**	21 (18–27)	26 (20–33)	0.01
**Shock**	34 (49%)	62 (65%)	0.04
**60-day Mortality**	14 (20%)	25 (26%)	0.36

### Continuous 11-gene score is associated with ICU mortality

**Figure 1:**
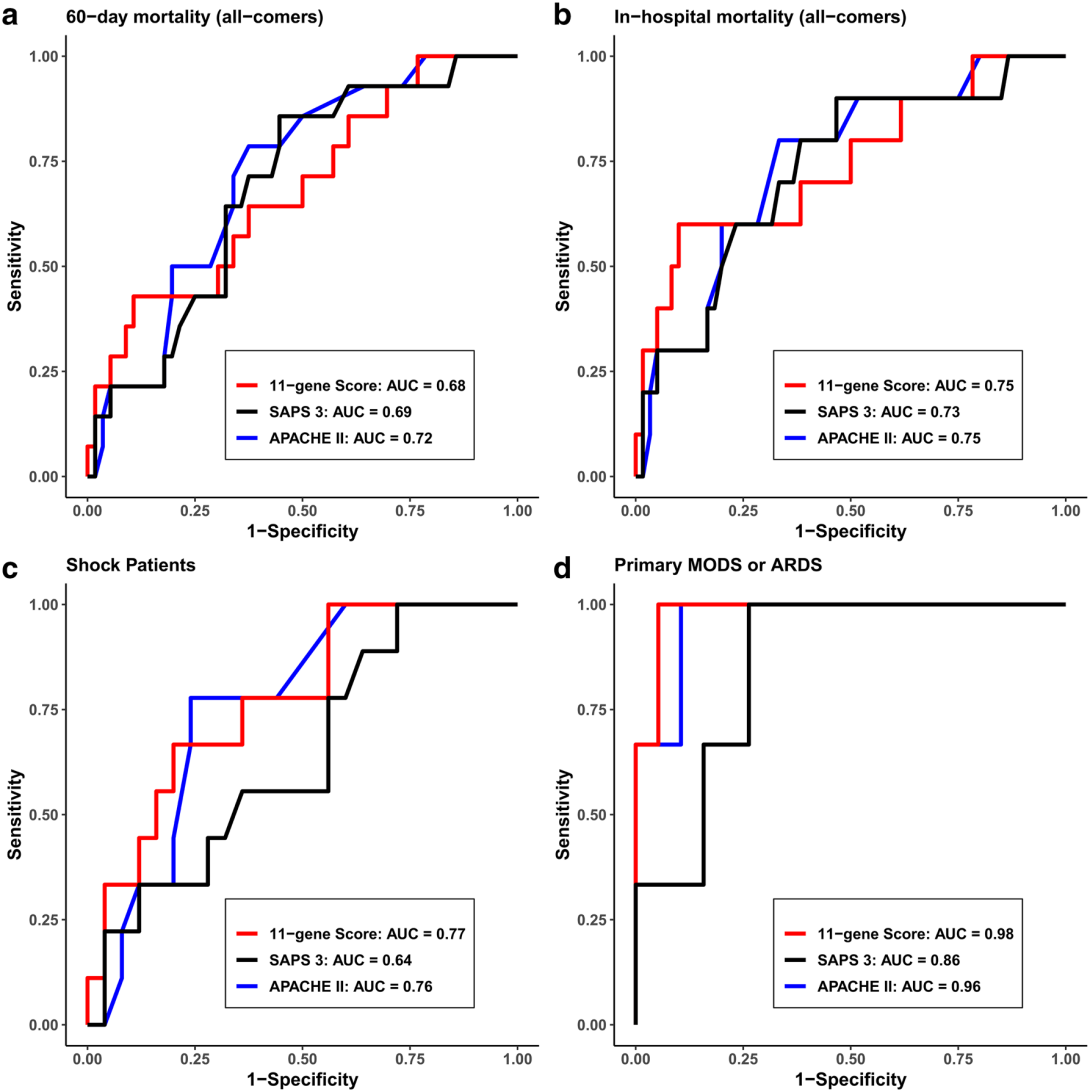
Receiver operating characteristic curves evaluating performance of the 11-gene score, SAPS 3, and APACHE II in patients with samples drawn within 24 h of emergency department arrival in predicting (**a**) 60-day mortality in all comers, (**b**) in-hospital mortality in all comers, (**c**) 60-day mortality in shock patients, and (**d**) 60-day mortality in the subgroup of patients with primary prognostic determinant of MODS and/or ARDS.

The 11-gene score obtained from a single blood draw is associated with 60-day mortality in subjects with samples obtained within 24 h of emergency department admission (AUROC 0.68, 95% CI 0.52–0.84). Performance was similar for SAPS 3 (AUROC 0.69, 95% CI 0.54–0.85) and APACHE II (AUROC 0.72, 95% CI 0.58–0.85) relative to the 11-gene score (Fig. 1a, p > 0.5). Additionally, the continuous 11-gene score was significantly associated with in-hospital mortality (AUROC 0.75, 95% CI 0.56–0.93, Fig. 1b), 30-day mortality (AUROC 0.73, 95% CI 0.57–0.91), and 60-day mortality in the pre-specified subgroup of patients with shock (defined as requiring one vasopressor) (AUROC 0.77, 95% CI 0.6–0.95, Fig. 1c). In all subgroups, performance of the 11-gene score remained similar to SAPS 3 and APACHE II (p > 0.25 in all subgroups). In all subgroups, performance of the continuous 11-gene score was poor in those patients who did not meet the 24 h emergency department cutoff, with AUROCs of 0.52–0.56.

As discussed above, each of the 70 patients with mRNA sampled within 24 h of emergency department presentation was clinician-phenotyped for primary driver of mortality (MODS or ARDS vs comorbidities or mixed presentation). The 11-gene mRNA score was highly accurate in patients with MODS or ARDS, with an AUROC of 0.98 (0.93–1, Fig. 1d). In contrast, performance was poor among subjects whose mortality is driven solely or substantially by underlying comorbidities or goals of care limitations, with an AUROC of 0.59 (0.4–0.79).

### Binary 11-gene score is associated with ICU mortality

**Figure 2:**
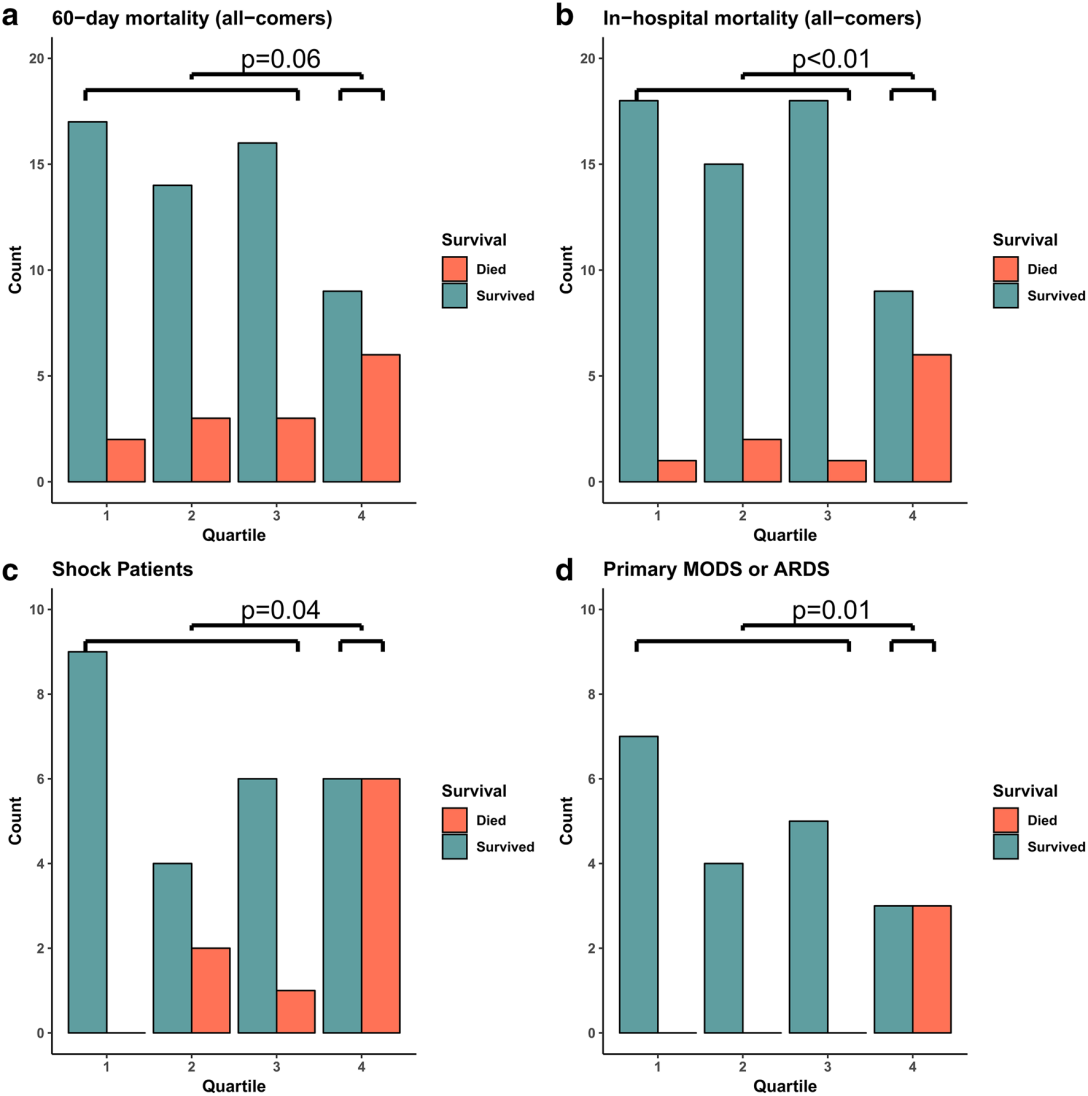
Bar graphs of outcomes by 11-gene score quartile in patients with samples drawn within 24 h of emergency department arrival evaluating (**a**) 60-day mortality in all comers, (**b**) in-hospital mortality in all comers, (**c**) 60-day mortality in shock patients, and (**d**) 60-day mortality in the subgroup of patients with primary prognostic determinant of MODS and/or ARDS. P-values are Fisher’s exact test comparting outcomes in top-quartile versus patients in bottom three quartiles.

**Figure 3:**
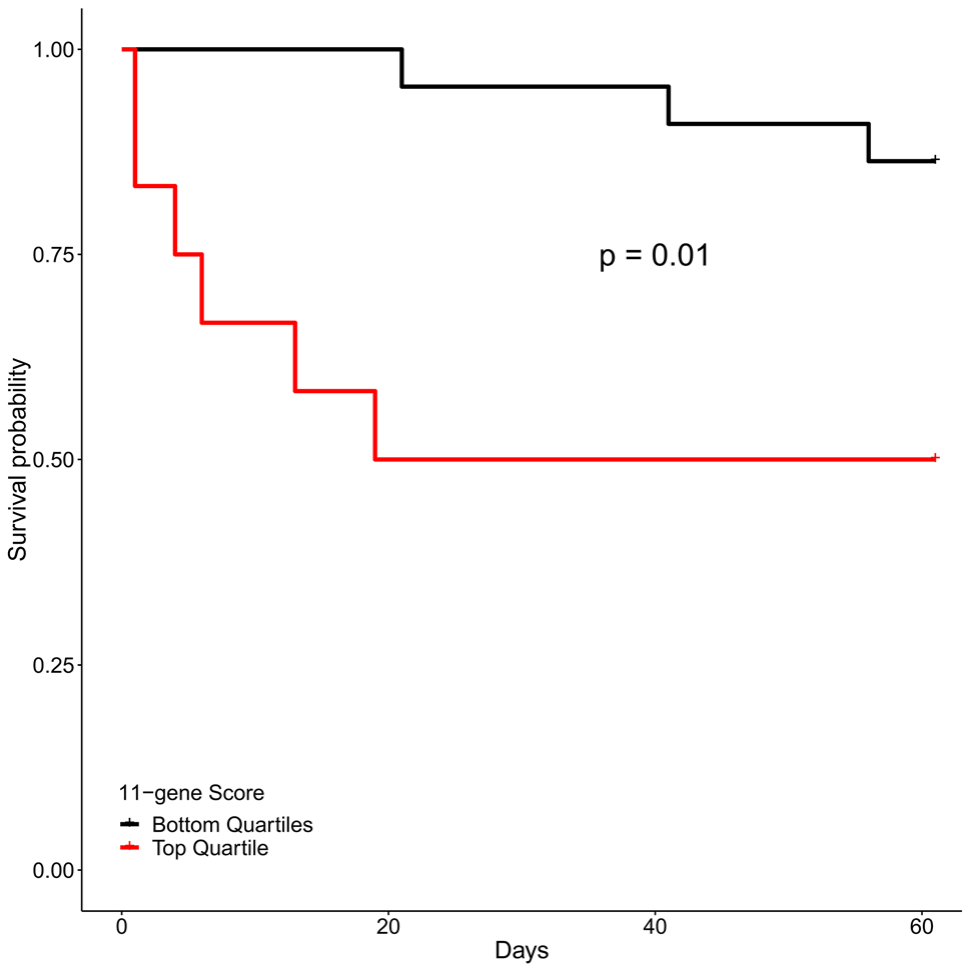
Kaplan–Meier curves for 60-day mortality in samples collected within 24 h of emergency department arrival (n = 70) separated by patients in the top quartile of 11-gene scores and those in the bottom three quartiles of gene scores. P-value was calculated using log-rank test.

To evaluate the utility of an easily interpretable cut-off, we dichotomized the results of the 11-gene score into pre-defined subgroups of the highest quartile vs. all others. The top quartile of scores in all samples had an 11-gene score greater than 4.02; 15 (21%) of the 70 subjects who met the 24 h emergency department cutoff had an 11-gene score above this threshold. Characteristics of these patients are summarized in Table 2. High 11-gene score subjects had a higher incidence of shock and higher APACHE II scores but were otherwise similar to their counterparts who did not meet the cutoff. The top quartile cutoff provided a sensitivity of 43% with a specificity of 84% in predicting 60-day mortality. Subjects in the highest 11-gene quartile experienced a trend towards a higher 60-day mortality at 40% vs 15% in all other quartiles with an odds ratio of 3.8 (0.9–16.6, Fisher’s Exact Test p = 0.06, Fig. 2a), and had a significantly higher risk of in-hospital mortality at 40% vs 7% in all others with an odds ratio of 8 (p < 0.01, Fig. 2b). In the pre-specified subgroup of patients with shock, patients in the top quartile experienced an odds ratio for 60-day death of 5.9 (p = 0.04, Fig. 2c). In those patients whose primary determinant of prognosis was MODS or ARDS, no patients with a low gene score died, while 50% of those with a score in the top quartile did (p = 0.01, Fig. 2d). Because the 11-gene score was derived to predict mortality in patients with acute sepsis, we hypothesized that the test would perform well in predicting early deaths but would perform poorly in predicting later deaths. To evaluate this further, we also performed Kaplan–Meier survival analysis for samples in the top quartile of 11-gene scores relative to samples in all other quartiles. Patients in the top quartile had significantly decreased survival relative to patients in the bottom quartiles (p = 0.01; Fig. 3).

**Table 2:** Characteristics of subjects in the top quartile vs bottom quartiles of 11-gene scores.

	Bottom quartiles* (n = 55)	Top quartile* (n = 15)	P-value
**Median age (IQR)**	71 (61–80)	67 (61–81)	0.88
**% Female**	38%	47%	0.57
**Race**			
White	39 (71%)	13 (87%)	0.32
Black/African American	4 (7%)	0 (0%)	0.57
Asian/Pacific Islander	5 (9%)	2 (13%)	0.64
Other/unknown	7 (13%)	0 (0%)	0.33
**Infection**	36 (65%)	11 (73%)	0.73
**Median SAPS 3 (IQR)**	59 (51–73)	64 (59–79)	0.14
**Median APACHE II (IQR)**	20 (17–25)	26 (21–31)	0.02
**Shock**	22 (40%)	11 (73%)	0.04
**Primary determinant of prognosis**			
MODS and/or ARDS	16 (29%)	6 (40%)	0.53
Mixed	20 (36%)	6 (40%)	1
Comorbidities	19 (35%)	3 (20%)	0.36
**60-day mortalit﻿y**	8 (15%)	6 (40%)	0.06

*Top quartile cutoff was calculated based on all 11-gene scores (all 165 patients).

### Addition of the 11-gene score adds prognostic value to existing severity of illness scores

**Figure 4:**
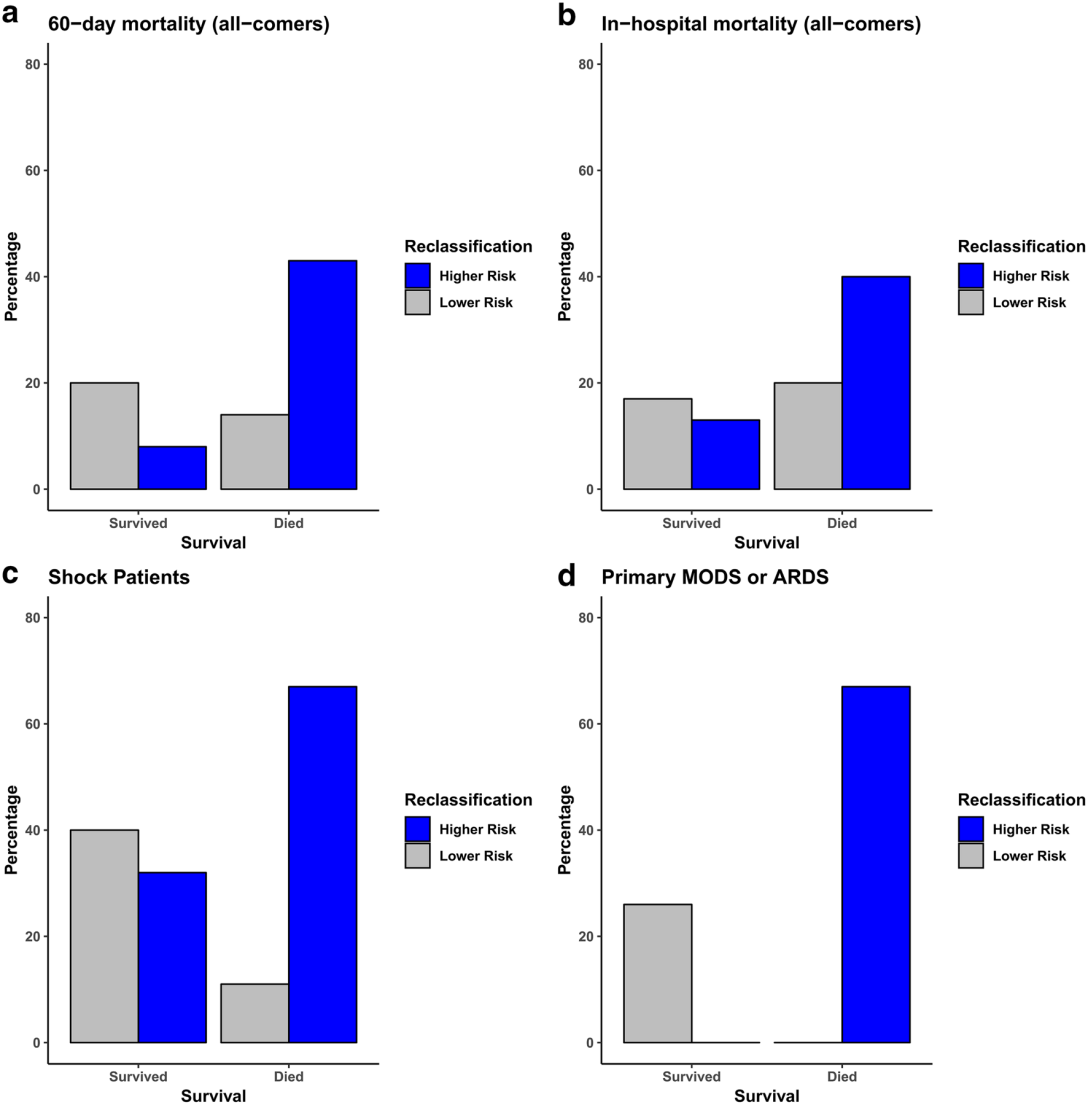
Bar graphs depicting the percentage of reclassifications into lower- and higher-risk groups when combining the 11-gene score with SAPS 3 using net reclassification improvement index in those patients with samples drawn within 24 h of emergency department arrival, evaluating (**a**) 60-day mortality in all comers, (**b**) in-hospital mortality in all comers, (**c**) 60-day mortality in shock patients, and (**d**) 60-day mortality in the subgroup of patients with primary prognostic determinant of MODS and/or ARDS. Correct reclassification is indicated by survivors who were reclassified to lower-risk group and deaths who were reclassified to high-risk group.

Addition of the continuous 11-gene score significantly improved risk stratification, with a categorical NRI of 0.45 (p = 0.03) and an IDI of 0.07 (p = 0.02) when combined with SAPS 3 and an NRI of 0.57 (p < 0.01) and IDI of 0.08 (p = 0.02) when combined with APACHE II (Table 3). To evaluate whether the 11-gene score added prognostic information in both low- and high-risk individuals we evaluated the percentage of correct and incorrect reclassifications when combined with SAPS 3 in those who died and survived for both our primary cohort as well as all subgroups, which is summarized in Fig. 4. In patients who survived, addition of the 11-gene score correctly reclassified 20% as lower risk and incorrectly reclassified 8% as higher risk. In patients who died, addition of the 11-gene score to SAPS 3 correctly reclassified 43% of patients as higher risk and incorrectly reclassified 14% of patients as lower risk. Performance was similar when combined with APACHE II and when using the binary gene-score cutoff (data not shown).

**Table 3:** AUROC, NRI, and IDI by subgroup.

Variable	11-gene score	SAPS 3	APACHE II
**24 h emergency department cutoff**			
AUROC (95% CI)	0.68 (0.52–0.84)	0.69 (0.54–0.83)	0.72 (0.58–0.85)
NRI (95% CI)		0.45* (0.05–0.84)	0.57** (0.22–0.93)
IDI (95% CI)		0.07* (0.01–0.14)	0.08* (0.01–0.15)
**Shock patients**			
AUROC (95% CI)	0.77 (0.6–0.95)	0.64 (0.43–0.85)	.76 (0.59–0.93)
NRI (95% CI)		0.64* (0.08–1.2)	0.5* (0.003–1)
IDI (95% CI)		0.2** (0.06–0.34)	0.23** (0.08–0.38)
**Primary determinant of prognosis is ARDS or MODS**			
AUROC (95% CI)	0.98 (0.93–1)	0.86 (0.67–1)	0.96 (0.88–1)
NRI (95% CI)		0.93** (0.36–1.5)	0.44 (− 0.11 to 0.99)
IDI (95% CI)		0.76** (0.39–1.12)	0.46* (0–0.92)

Performance as measured by AUROC in predicting 60-day mortality of the 11-gene score, SAPS3, and APACHE II are outlined above. Additionally, the categorical Net-Reclassification (NRI) Index and Integrated Discrimation Improvement (IDI) Index are shown for the comparison of SAPS3 and APACHE II alone vs in combination with the 11-gene score using logistic regression modeling. NRI and IDI values greater than 0 are suggestive of improved prognostic performance with the addition of the 11-gene score.

*P < 0.05.

**P < 0.01.

## Discussion

In this study, we prospectively validated the previously described 11-gene mortality score in a heterogeneous, real-world ICU population. This study showed that the 11-gene score performs similarly to SAPS 3 and APACHE II as a stand-alone test and when collected early in an emergency department admission. Importantly, our results strongly suggest that 11-gene mortality score provides additional prognostic information when combined with existing clinical scores. Patients in the highest quartile were nearly four times more likely to die in sixty days, showing that the 11-gene score may be amenable to pre-defined cutoffs that provide easily interpretable, actionable results. Additionally, as a gene expression panel, it is being worked into a broader mRNA expression panel for use as a rapid *in-vitro* diagnostic test with a 30-min turnaround time, potentially enabling earlier triage and clinical trial enrollment than currently available decision tools^24^.

This analysis builds on prior work in important ways. First, while some of the publicly available sepsis datasets in which the mRNA score was derived include patients recruited > 10 years ago, this is a modern cohort and reflects current standard of care delivery. Similarly, prior cohorts were more homogeneous septic populations (some included only bacterial sepsis, or H1N1-positive flu, for example). In contrast, this cohort includes an unselected ICU population containing a substantial portion of patients in whom sepsis was uncertain or only probable. The diagnosis of sepsis is often unclear at presentation, and a score that functions well despite diagnostic uncertainty is valuable. For both of these reasons, the fact that the 11-gene mortality score maintained performance with an AUROC comparable to more complex scoring systems is important.

This work also underscores the highly dynamic nature of gene expression in acute illness, and reinforces prior research demonstrating that timing of blood draw is critical. Sweeney et al*.* had previously found in the surgical populations collected for the Glue Grant that an initial inflammatory signal is replaced within 24-48 h with a distinct pattern associated with recovery^21^. The present study supports this finding, with the 11-gene mortality score achieving excellent discrimination early in the course of illness, while performing poorly in patients who were sampled later (> 24 h from emergency department presentation).

A major issue in ICU clinical trials is the heterogeneity of ICU populations and the competing risks of death. Iwashyna and colleagues have reported in population data of millions of individuals that widely-used mortality scores (e.g. APACHE and SAPS scores) are prognostic in the first 10 days, but beyond that, mortality is driven by underlying illness^25^. As a result, there has been significant interest in identifying biomarkers that identify high risk subgroups for predictive and prognostic enrichment of trials^14,26,27^. We hypothesized that the 11-gene score would work well in patients whose mortality was driven by acute illness, and less well in patients in whom chronic illness was a substantial driver of mortality. To test this hypothesis, physicians phenotyped each patient for key drivers of mortality, finding that the 11-gene score indeed works extremely well in the subset of patients whose prognosis was primarily driven by acute illness, while working poorly in those in those whom comorbidities (e.g. metastatic cancer, progressive ALS, or cirrhosis) or goals of care were a major driver of prognosis. This disparity is likely to be a limitation to any ICU prognostic biomarker of acute inflammation or organ failure, as such patients are unlikely to have the same underlying pathophysiology.

This study has several limitations. First, sample size is small, with only 70 patients with specimens obtained within 24 h of emergency department presentation. Although the score works well in those patients, they represent less than half of the subjects recruited into the ICU biobank. The 11-gene score is unlikely to work well for a substantial fraction of ICU patients admitted from the wards or as transfers from outside hospitals because of issues of dynamic and rapidly evolving mRNA signals; this same group is under-represented in most biobanks and clinical trials populations, and merits further study. Stanford is a tertiary hospital with a very high proportion of immune-suppressed and chronically ill patients, raising issues of generalizability. Less than a third of the population was identified as having mortality driven primarily by acute illness rather than underlying comorbidities. Nonetheless, the score worked extremely well in these patients (AUROC 0.98, mortality in the high-mRNA quartile 50% vs 0% in all other quartiles), suggesting that performance of the mortality score may have been biased to the null in our Stanford cohort. Finally, the threshold of 4 for highest quartile was derived within our population. To be widely adopted, the performance needs to be further evaluated with a set cut-point in additional populations.

## Data Availability

The datasets used and/or analyzed during the current study are available from the corresponding author on reasonable request.
